# Associations of corticosteroid therapy and tonsillectomy with kidney survival in a multicenter prospective study for IgA nephropathy

**DOI:** 10.1038/s41598-023-45514-4

**Published:** 2023-10-27

**Authors:** Tetsuya Kawamura, Keita Hirano, Kentaro Koike, Masako Nishikawa, Akira Shimizu, Kensuke Joh, Ritsuko Katafuchi, Akinori Hashiguchi, Keiichi Matsuzaki, Shoichi Maruyama, Nobuo Tsuboi, Ichiei Narita, Yuichiro Yano, Takashi Yokoo, Yusuke Suzuki

**Affiliations:** 1 Division of Nephrology and Hypertension, Department of Internal Medicine, The Jikei University School of Medicine, 3-25-8 Nishi-Shinbashi, Minato-Ku, Tokyo, 105-8461 Japan; 2 Division of Nephrology, Department of Internal Medicine, Japanese Red Cross Ashikaga Hospital, Ashikaga, Japan; 3 Clinical Research Support Center, The Jikei University School of Medicine, Tokyo, Japan; 4 Department of Analytic Human Pathology, Nippon Medical School, Tokyo, Japan; 5 Department of Pathology, The Jikei University School of Medicine, Tokyo, Japan; 6 National Hospital Organization Fukuoka-Higashi Medical Center, Fukuoka, Japan; 7 Division of Nephrology, Department of Internal Medicine, Kano Hospital, Fukuoka, Japan; 8 Department of Pathology, Keio University School of Medicine, Tokyo, Japan; 9 Department of Public Health, Kitasato University School of Medicine, Sagamihara, Kanagawa Japan; 10 Department of Nephrology, Nagoya University Graduate School of Medicine, Nagoya, Japan; 11 Division of Clinical Nephrology and Rheumatology, Niigata University Graduate School of Medical and Dental Sciences, Niigata, Japan; 12 Department of Advanced Epidemiology, Noncommunicable Disease (NCD) Epidemiology Research Center, Shiga University of Medical Science, Ōtsu, Shiga Japan; 13 The Department of Family Medicine and Community Health, Duke University, Durham, NC USA; 14 Department of Nephrology, Juntendo University Faculty of Medicine, Tokyo, Japan

**Keywords:** Medical research, Nephrology

## Abstract

Efficacy of systemic corticosteroid therapy (CS) for long-term kidney survival in patients with IgA nephropathy (IgAN) is controversial. Therefore, prospective studies evaluating targeted therapies to lymphatic tissues in mucosal immune system responsible for production of nephritogenic IgA have been desired worldwide. Here, we aimed to evaluate the associations of CS and combination therapy of CS and tonsillectomy (CS + Tx) with kidney survival, using database from a nationwide multicenter prospective cohort study on IgAN. Primary outcome was a 50% increase in serum creatinine from baseline or dialysis induction. The analysis included 941 patients (CS/CS + Tx/non-CS 239/364/338), 85 (9.0%) of whom reached outcomes during median follow-up of 5.5 (interquartile range 2.0–8.0) years. On overlap weighting analysis with balanced baseline characteristics, CS and CS + Tx were associated with lower risk of kidney events when compared with non-CS (hazard ratio [HR] 0.51, 95% confidence interval [CI] 0.29–0.88 and HR 0.20, 95%CI 0.09–0.44, respectively). Notably, when compared with the CS, CS + Tx was associated with a lower risk of kidney events (HR 0.40, 95%CI 0.18–0.91). Present study demonstrated, keeping with favorable association of systemic CS with kidney survival, concurrent tonsillectomy as one of targeted interventions to lymphatic tissues may provide additional improvement to kidney survival in patients with IgAN.

## Introduction

Immunoglobulin A nephropathy (IgAN) is the most common type of glomerulonephritis worldwide, and 30–40% of affected patients develop end-stage kidney disease (ESKD) within 20 years from its onset^1^. The KDIGO international clinical practice guideline recommends the systemic administration of corticosteroids (CS) in patients with IgAN who remain at high risk of ESKD despite maximal supportive care^2–7^. Nevertheless, the effect of CS on kidney events among patients with IgAN has been controversial^2,3,6,8–11^. Lv et al. demonstrated the efficacy of CS on kidney survival by the largest randomized controlled study (RCT) for IgAN^4,5^. On the contrary, Rauen et al. found no differences in kidney survival in IgAN patients randomized to receive added CS on top of supportive care compared to supportive care alone^3,12^. In addition, adverse effects of CS which cannot be overlooked were observed in some studies^3–5^. Therefore, new strategies improving the efficacy and safety of CS have been desired worldwide^13^.

Targeted therapies to lymphatic tissues in mucosal immune system responsible for production of nephritogenic IgA may improve kidney survival in patients with IgAN^14–20^. Mucosa-associated lymphoid tissue includes gut-associated lymphoid tissue (GALT) and nasal/upper respiratory mucosa-associated one (NALT). Regarding GALT, an enteric targeted-release formulation of budesonide excellently reduced proteinuria in patients with IgAN in the recent NEFIGAN trial^14^. The long-term kidney survival on budesonide treatment may be clarified elsewhere. Gross hematuria following acute upper respiratory infections and COVID-19 may indicate the apparent association between NALT and IgAN^21,22^. Compared to CS alone, reduction in proteinuria by tonsillectomy combined with CS (CS + Tx) has been demonstrated in two RCTs from East Asia^15,16^. A meta-analysis in 2017, our recent matched study in 2019 and other retrospective studies after 2020 reported an association between tonsillectomy and kidney survival^17,18,23,24^. However, these two studies were retrospective in nature and did not include an assessment of wide spectrum baseline clinicopathological characteristics. Therefore, further clinical implications of CS + Tx should be clarified by long-term prospective observational studies.

Using data from the nationwide prospective cohort study (Japan IgA Nephropathy Prospective Cohort Study [J-IGACS])^25^, the current study assessed associations of CS and CS + Tx with kidney survival compared to non-corticosteroid use (non-CS) and further compared CS + Tx with CS on kidney survival.

## Results

### Study cohort and kidney outcomes

**Figure 1:**
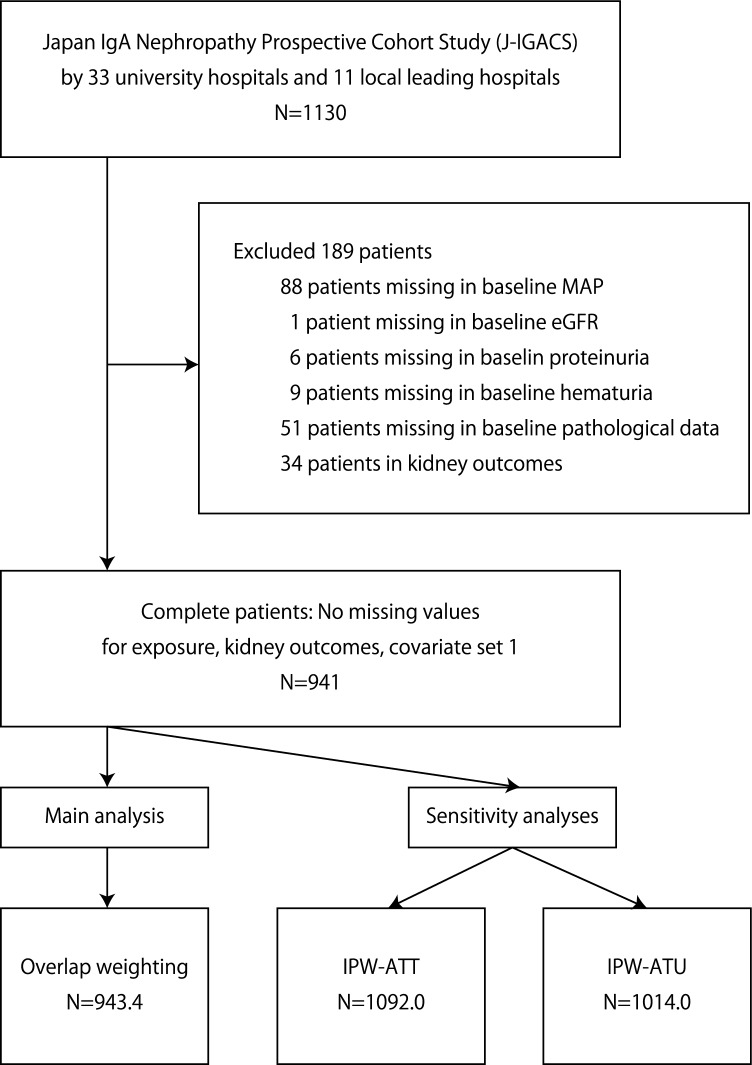
Patient flowchart. MAP, mean arterial blood pressure; eGFR, estimated glomerular filtration rate; IPW-ATT, inverse probability treatment weighting on the average treatment effect in the population that received CS + Tx; IPW-ATU, inverse probability treatment weighting on the average treatment effect in the non-CS group.

In total, 1130 participants were registered in J-IGACS spanning 33 university hospitals and 11 local leading hospitals in Japan (Fig. 1, Supplementary Table S1). After excluding 88 patients with a missing mean arterial blood pressure (MAP) measurement, 1 with a missing estimated glomerular filtration rate (eGFR) measurement, 6 with a missing proteinuria measurement, and 9 with a missing hematuria measurement at the index date, 51 patients missing kidney biopsy data and 34 with missing data regarding outcomes, the final analysis set comprised 941 participants (Fig. 1, covariate set 1 in Table 1, covariate set 2 in Supplementary Table S2). The median baseline hematuria (number of urinary sediment red blood cells [uRBC]/high power filed [HPF]) and MAP in these 941 patients was 20/HPF and 88.7 mmHg, respectively. Therefore, they were categorized into two groups: uRBC > 20/HPF and uRBC ≤ 20/HPF, and ≥ 90 mmHg and < 90 mmHg, respectively. During median follow-up of 5.5 (interquartile range [IQR] 2.0–8.0) years, 85 (9.0%) of the 941 patients reached the outcomes.

**Table 1:** Baseline characteristics in covariate set 1 according to the initial treatment categories by unweighted and overlap-weighted analysis.

Baseline characteristics	Unweighted				Overlap-weighted			
	non-CS	CS	CS + Tx	MASD	non-CS	CS	CS + Tx	MASD
No. of patients	338	239	364		314.5	314.5	314.5	
Age, years	42.3 ± 17.3	40.9 ± 19.1	34.8 ± 13.4	0.49	40.3 ± 16.6	40.2 ± 18.3	38.0 ± 14.3	0.15
Age < 20 years^a^	26 (7.7)	32 (13.4)	43 (11.8)	0.19	27.9 (8.9)	38.8 (12.3)	27.6 (8.8)	0.12
Woman	162 (47.9)	117 (49.0)	200 (54.9)	0.14	155.7 (49.5)	157.2 (50.0)	165.8 (52.7)	0.06
DM	5 (1.5)	9 (3.8)	6 (1.6)	0.14	7.9 (2.5)	5.6 (1.8)	10.7 (3.4)	0.10
MAP, mmHg	91.1 ± 13.6	91.9 ± 14.5	88.0 ± 13.3	0.28	90.9 ± 13.7	91.0 ± 14.2	89.9 ± 13.7	0.08
MAP ≥ 90 mmHg^a^	179 (53.0)	121 (50.6)	144 (39.6)	0.27	169.0 (53.7)	157.6 (50.1)	146.1 (46.5)	0.15
eGFR, ml/min/1.73 m^2^	73.2 ± 28.9	71.2 ± 31.0	80.7 ± 27.0	0.33	73.0 ± 28.0	73.3 ± 29.8	76.3 ± 26.3	0.12
eGFR < 60 ml/min/1.73 m^2a^	119 (35.2)	82 (34.3)	86 (23.6)	0.26	100.6 (32.0)	104.0 (33.1)	87.0 (27.7)	0.12
Proteinuria, g/day	0.38 (0.18–0.81)	0.88 (0.45–1.81)	0.62 (0.3–1.16)	0.64	0.58 (0.25–1.18)	0.65 (0.39–1.30)	0.57 (0.26–1.14)	0.09
Proteinuria > 1.0^a^	70 (20.7)	109 (45.6)	109 (29.9)	0.55	105.8 (33.6)	110.2 (35.0)	92.6 (29.4)	0.12
Hematuria ≥ 20/HPF	149 (44.1)	137 (57.3)	205 (56.3)	0.27	162.9 (51.8)	165.0 (52.5)	160.4 (51.0)	0.03
RAASi use	210 (62.1)	135 (56.4)	191 (52.5)	0.20	188.8 (60.0)	186.6 (59.4)	176.4 (56.1)	0.08
MEST-C score								
M1	82 (24.2)	92 (38.5)	100 (27.5)	0.31	98.3 (31.3)	100.5 (32.0)	96.3 (30.6)	0.03
E1	74 (21.9)	121 (50.6)	139 (38.2)	0.63	118.8 (37.8)	124.9 (39.7)	118.3 (37.6)	0.04
S1	214 (63.3)	195 (81.6)	286 (78.6)	0.42	238.3 (75.8)	242.8 (77.2)	233.7 (74.3)	0.07
T1 + 2	76 (22.5)	64 (26.8)	66 (18.1)	0.21	69.5 (22.1)	76.9 (24.5)	63.4 (20.2)	0.10
C1 + 2	72 (21.3)	124 (51.9)	160 (46.4)	0.67	122.4 (38.9)	131.4 (41.8)	114.4 (36.4)	0.11

Unweighted and weighted values are shown as number (%), mean ± SD, or median (IQR). DM was defined as physician diagnosis of diabetes or antihyperglycemic medication use at time of kidney biopsy. The number (%) of patients who underwent only tonsillectomy within one year after kidney biopsy in the non-CS group was 42 (12.4) before weighted analysis and 46 (13.5) after weighted analysis.

non-CS, non-corticosteroid therapy; CS, corticosteroid monotherapy; CS + Tx, corticosteroid combined with tonsillectomy; MASD, max of the pairwise absolute standardized difference; DM, diabetes mellitus; MAP, mean arterial blood pressure; eGFR, estimated glomerular filtration rate; RAASi, renin–angiotensin–aldosterone system inhibitor; M, mesangial hypercellularity score; E, endocapillary hypercellularity score; S, segmental sclerosis score; T, tubulointerstitial fibrosis/atrophy score; C, crescent score; ND, not determined.

^a^Neither included in covariate set 1 nor employed for propensity score but shown here to promote understanding of the cohort.

### Associations of corticosteroid therapy and concurrent tonsillectomy with kidney outcomes

**Figure 2:**
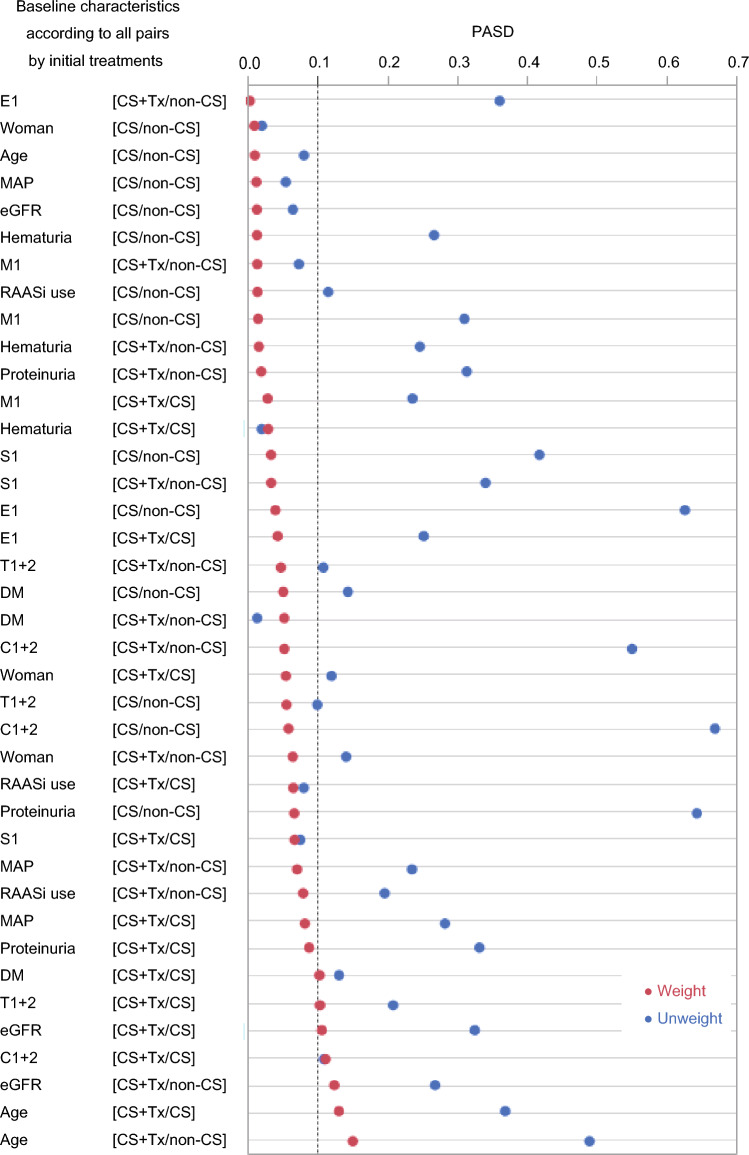
All pairwise absolute standardized differences (PASDs) in baseline characteristics among the initial treatment categories before and after overlap-weighting analysis. Distribution of all baseline characteristics in covariate set 1 according to the treatment groups (non-CS, CS, and CS + Tx) was balanced after overlap-weighting analysis as shown by PASD. The rate of variables showing PASD ≤ 0.1 in covariate set 1 increased from 25.6% in the unweighted analysis to 87.2% in the weighted analysis. The maximums of the pairwise absolute standardized differences in each baseline characteristic are also shown in Table 1. PASD, all pairwise absolute standardized difference; non-CS, non-corticosteroid use; CS corticosteroid monotherapy; CS + Tx, corticosteroid therapy combined with tonsillectomy; MAP, mean arterial blood pressure; eGFR, estimated glomerular filtration rate; M, mesangial hypercellularity score; E, endocapillary hypercellularity score; S, segmental sclerosis score; T, tubulointerstitial fibrosis/atrophy score; C, crescent score; RAASi, renin–angiotensin–aldosterone system inhibitor.

**Figure 3:**
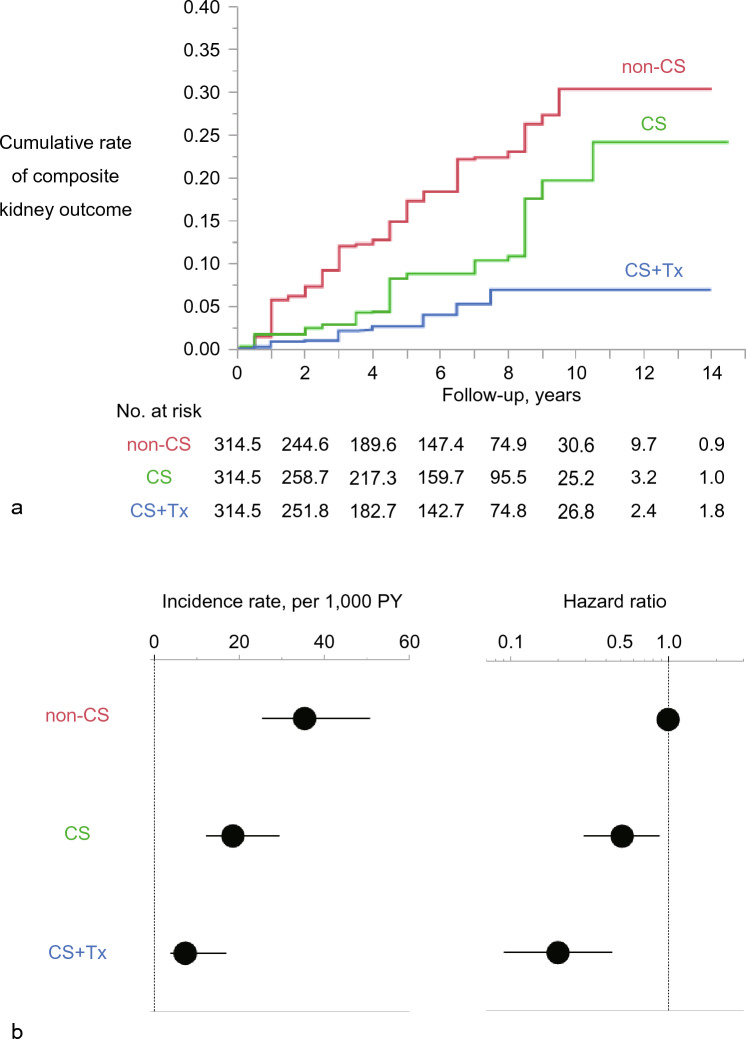
Cumulative incidence estimates, incidence rate, and hazard ratio for kidney outcome in the overlap-weighting analysis. (**a**) Weighted cumulative incidence estimates by initial treatment categories. The date of kidney biopsy was considered as the index date for all patients. In overall, composite kidney events were defined as > 50% increase in serum creatinine from baseline (among patients aged ≥ 20 years; the number of events [%] was 96.6/849.1 [11.4] in the overlap-weighting analysis) or > 25% reduction in eGFR (among patients aged < 20 years; the number of events [%] was 5.4/94.3 [5.7] in the overlap-weighting analysis). These definitions were used because all patients who received maintenance dialysis during follow-up reached these end points prior to the induction of dialysis. The median observation time by initial treatment categories were 5.5 years (IQR, 2.0–7.5 years) in the non-CS group, 6.0 years (IQR, 3.0–8.0 years) in the CS group, and 5.0 years (IQR, 2.0–7.5 years) in the CS + Tx group. During follow-up, the median initiated time of the treatments was 0.0 years (IQR, 0.0–0.0 years) in the non-CS group, 0.0 years (IQR, 0.0–0.5 years) in the CS group, and 0.0 years (IQR, 0.0–0.5 years) in the CS + Tx group. (**b**) Weighted incidence rate per 1000 patient-years and weighted hazard ratio by initial treatment categories. Actual data are shown in Table 2. PY, patient-years; HR, hazard ratio; CI, confidence interval; non-CS, non-corticosteroid use; CS, corticosteroid monotherapy; CS + Tx, corticosteroid combined with tonsillectomy.

After performing an overlap weighting analysis (Fig. 1), the distribution of baseline covariates among the initial treatment categories (non-CS, CS, and CS + Tx) was well-balanced (Table 1, Fig. 2, Supplementary Table S2). Overlap-weighted incidence of composite kidney events was significantly lower in the CS group compared to the non-CS group (Fig. 3 and Table 2). CS was also associated with a lower risk of composite kidney events compared with non-CS. Notably, CS + Tx was associated with a lower risk of composite kidney events compared to non-CS and CS. As demonstrated in Fig. 3, a stepwise reduction in risk of kidney outcomes was observed from the non-CS group to the CS group, followed by the CS + Tx group.

**Table 2:** Associations between initial treatments and composite kidney outcomes in overlap-weighted analysis, IPW-ATT analysis, and IPW-ATU analysis.

Weighting	Initial treatment	Incidence rate per 1,000 PY (95% CI)	non-CS as reference				CS as reference			
			HR (95% CI)	*p* value	E value for point estimate	E value for 95% CI	HR (95% CI)	*p* value	E value for point estimate	E value for 95% CI
Overlap (main analysis)	non-CS	35.4 (25.3–50.7)	1.0	Reference						
	CS	18.5 (12.1–29.4)	0.51 (0.29–0.88)	0.015	3.35	1.54	1.0	Reference		
	CS + Tx	7.3 (3.7–16.9)	0.20 (0.09–0.44)	< 0.001	9.34	3.94	0.40 (0.18–0.91)	0.029	4.45	1.43
IPW-ATT	non-CS	41.9 (25.6–74.2)	1.0	Reference						
	CS	13.9 (8.5–24.1)	0.32 (0.16–0.65)	0.001	5.71	2.47	1.0	Reference		
	CS + Tx	5.7 (3.2–11.1)	0.13 (0.06–0.29)	< 0.001	14.6	6.44	0.41 (0.19–0.90)	0.025	4.27	1.48
IPW-ATU	non-CS	26.8 (20.3–36.1)	1.0	Reference						
	CS	24.7 (14.2–47.4)	0.88 (0.48–1.62)	0.681	1.53	1.00	1.0	Reference		
	CS + Tx	6.0 (2.7–15.8)	0.22 (0.10–0.52)	< 0.001	8.47	3.30	0.25 (0.10–0.65)	< 0.001	7.39	2.45

IPW, inverse probability treatment weighting analysis; ATT, average treatment effect in the treated as CS + Tx group; ATU, average treatment effect in the untreated non-CS group; PY, patient-years; CI, confidence interval; HR, weighted hazard ratio; non-CS, non-corticosteroid use; CS, corticosteroid monotherapy; CS + Tx, corticosteroid therapy combined with tonsillectomy.

### Interactions between baseline characteristics and the initial treatment for kidney outcomes

**Figure 4:**
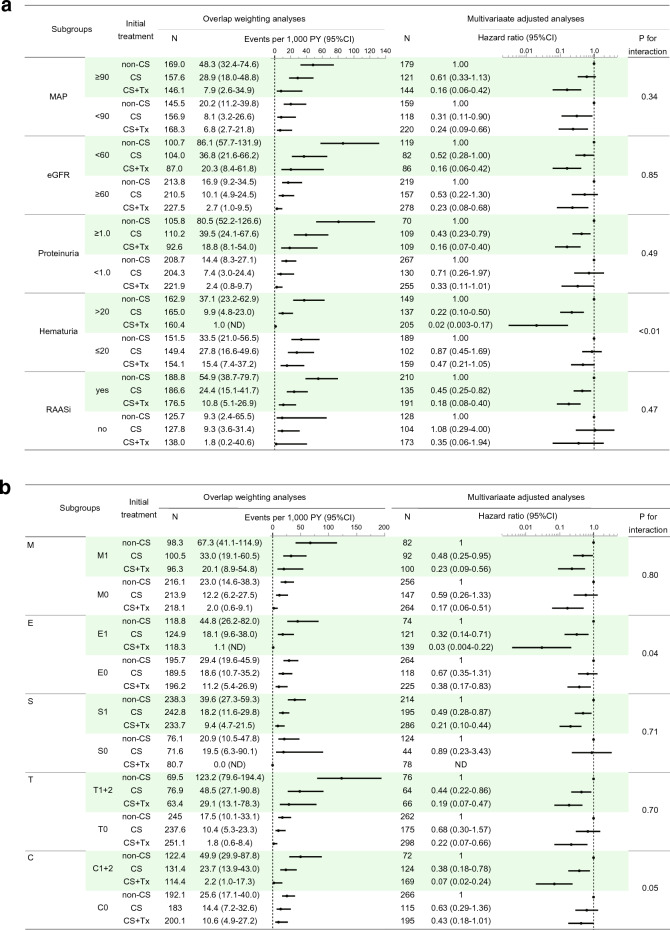
Stepwise reduction in risk of kidney outcomes, in order, by non-corticosteroid use, corticosteroid monotherapy, and combining corticosteroid with tonsillectomy according to baseline characteristic subgroups. The association between initial treatments and kidney outcomes (already shown in Fig. 3 and Table 2) was not modified by a majority of the baseline characteristic subgroups (*p* for interaction > 0.1). (**a**) Clinical subgroup analyses. One significant interaction between hematuria and initial treatment was observed. (**b**) Pathological subgroup analyses. Two significant interactions between each of endocapillary hypercellularity and crescent and initial treatment were observed. Hazard ratios were not weighted but adjusted with all variables in covariate set 1, except for each stratifying factor. N, number of patients; PY, weighted patient-years; CI, confidence interval; P for interaction, *p* value for interaction between initial treatments and each of the baseline characteristic subgroups on kidney outcome; non-CS, non-corticosteroid use; CS, corticosteroid monotherapy; CS + Tx, corticosteroid therapy combined with tonsillectomy; MAP, mean arterial pressure; eGFR, estimated glomerular filtration rate; RAASi, renin–angiotensin–aldosterone system inhibitor; M, mesangial hypercellularity; E, endocapillary hypercellularity; S, segmental sclerosis; T tubular atrophy/interstitial fibrosis; C, crescent; ND, not determined because of small sample size.

The associations between initial treatments and kidney outcomes described above were not affected by almost any baseline characteristic stratifications with explored overlap weighting analyses and multivariate adjusted analyses (Fig. 4). In these subgroup analyses, a gradual decrease in the risk of kidney outcomes from the non-CS group to the CS group, and then to the CS + Tx group, was consistently observed. For instance, those favorable associations were found in both higher proteinuria (≥ 1.0 g/day) and lower proteinuria (< 1.0 g/day) subgroups. In addition, three significant interactions on kidney outcomes were identified, which were between the initial treatment and each of the following baseline characteristics: microscopic hematuria (Fig. 4a), endocapillary hypercellularity, and crescent (Fig. 4b). In patients with greater microscopic hematuria, a more pronounced decrease in the risk of kidney outcomes was observed from the non-CS group to the CS group, followed by the CS + Tx group (Fig. 4a). Conversely, patients with lesser baseline hematuria had worse treatment outcomes. No significant risk reduction was observed in either the CS or CS + Tx group compared to the non-CS group. Similar results were seen in endocapillary hypercellularity (E1/E0) and crescent (C1 + 2/C0) subgroups.

### Sensitivity analyses

The inverse probability treatment weighting on the average treatment effect in the population that received CS + Tx (IPW-ATT) cohort had the highest frequency of patients with greater hematuria, E1 and C1 + 2, followed by the overlap-weighting and inverse probability treatment weighting on the average treatment effect in the non-CS group (IPW-ATU) cohorts (Supplementary Table S3). Conversely, the highest rate of adverse predictors commonly observed in patients with chronic kidney disease (e.g., MAP ≥ 90 mmHg and eGFR < 60 ml/min/1.73 m^2^) was seen in the IPW-ATU cohort, followed by the overlap-weighting and IPW-ATT cohorts.

A weighted Cox regression model applied to the IPW-ATT cohort demonstrated the robustness of the association between the initial treatment categories and kidney outcomes (Table 2). CS and CS + Tx were associated with a lower risk of kidney events compared to non-CS. CS + Tx was further associated with a lower risk of kidney events compared to CS. In contrast, the IPW-ATU analysis did not show any significant difference in kidney outcomes between the CS and non-CS groups. However, CS + Tx was still associated with a lower risk of kidney events compared to CS and non-CS. The *E*-values indicated that unmeasured confounders were unlikely to sufficiently attenuate the main finding shown in Fig. 3 and Table 2 and negate a causal relationship between corticosteroid treatments and kidney outcomes.

## Discussion

Overlap propensity score analysis in the present study achieved good balance among patients with IgAN subjected to different initial treatments and demonstrated that corticosteroid monotherapy and concurrent tonsillectomy were independently associated with better kidney outcomes. Those associations were especially pronounced in patients with endocapillary hypercellularity, crescent, and greater microscopic hematuria at baseline. These results may be of clinical significance in facilitating better identification of subgroups of patients with IgAN who will benefit from corticosteroid therapy and tonsillectomy, and in avoiding potential overtreatment^2^.

Our results demonstrating the association of corticosteroid monotherapy with better kidney outcomes are in line with the findings of previous studies (Fig. 3 and Table 2)^6,7,26^. Zhang et al. reported a meta-analysis reviewing six RCTs published between 1993 and 2017^26^. The meta-analysis revealed that the use of corticosteroids can significantly decrease the risk for ESKD. Two observational studies matched by propensity score, the VALIGA and Tokyo cohorts, also confirmed the association of corticosteroid therapy with better kidney survival^6,7^. In contrast, the STOP-IgAN trial compared immunosuppression based on corticosteroids with standard care but did not find kidney benefits^3^. The reasons for these contradictory findings are not clear but may be related to the heterogeneity of MEST-C scores (Supplementary Table S4) and patient clinical characteristics. For example, the occurrence of endocapillary hypercellularity and crescent in STOP-IgAN appeared to be lower than that in most other studies reporting an advantage of corticosteroid treatment in kidney survival. Our sensitivity analysis with the IPW-ATU cohort also had lower occurrence of endocapillary hypercellularity and crescent (Supplementary Table S3) and found no significant difference in kidney outcomes between the CS and non-CS groups. Different prognoses and/or therapeutic responses have been proposed among various races or ethnicities. Barbour et al. described in a Canadian observational study that persons of Pacific Asian origin might be at an increased risk of disease progression^27^.

The favorable association between corticosteroid use and kidney outcomes was greater in patients in the CS + Tx group than in the CS group (Fig. 2 and Table 2). Two RCTs from East Asia demonstrated a significant antiproteinuric effect of CS + Tx compared with CS^15,16^. A multicenter matched study in Japan revealed the association of tonsillectomy with better kidney survival independently from corticosteroid use^17^. The recent international clinical guideline added a special recommendation of tonsillectomy for Japanese patients with IgAN receiving corticosteroid therapy^2^. In contrast, the association of tonsillectomy with better kidney survival was not confirmed in European studies^28,29^. The reasons for these discrepant findings are not clear, but may relate to patient characteristics and the sample size of patients who underwent tonsillectomy. A genome-wide association study from Danish health registries demonstrated the association of one genetic variant with tonsillectomy risk (owing to severe tonsillitis or massive tonsillar hypertrophy) and IgAN susceptibility^30^. The same variant was also associated with IgAN progression in other studies^31,32^. Similar to the recent NEFIGAN trial, which was designed to deliver an enteric targeted-release formulation of budesonide to the distal ileum in patients with IgAN and demonstrated a significant antiproteinuric effect of the drug, tonsillectomy may be associated with better kidney outcome as one of the targeted therapies to lymphatic tissues responsible for production of nephritogenic IgA^14^. Recent findings from basic research indicate that tonsillar B lymphocytes or nasal-associated lymphoid tissue play a pivotal role in the production of nephritogenic IgA1, providing substantial support for this hypothesis^33,34^. In a similar vein, Kawabe et al. reported that conducting a tonsillectomy one year after kidney transplantation was correlated with a reduced incidence of histological recurrence of IgAN^35^. Further studies are needed to clarify how overtreatment with CS + Tx can be reduced, as the present study demonstrates that CS alone is associated with lower adverse kidney outcomes compared with non-CS treatment.

As indicated in KDIGO Guideline 2021, the present study found a significant association between initial treatment and kidney outcome among individuals with severe proteinuria (≥ 1.0 g/day). Meanwhile, there was no significant interaction observed between the proteinuria level (≥ 1.0 g/day and < 1.0 g/day) and the type of initial treatment (non-CS/CS/CS + Tx), as depicted in Fig. 4a. This observation aligns with findings from prior studies^17,36,37^. It is worth emphasizing that physicians should not hesitate unduly to administer corticosteroid treatment (+/− tonsillectomy) to patients with lower proteinuria than 1.0 g/day, in addition to providing supportive care. This perspective is substantiated by a study from China, which reported that patients with time-averaged proteinuria levels ranging from 0.5 to 1.0 g/day exhibited worse kidney outcomes^38^. However, it is crucial to highlight that our findings should be validated through further extensive observational studies and RCTs to establish their reliability.

The association of corticosteroid monotherapy with better kidney outcomes was not observed in patients with lesser baseline hematuria but in those with greater baseline hematuria in a subgroup analysis (Fig. 4a). To the best of our knowledge, this is the first study to focus on the different associations between corticosteroid use and kidney outcomes based on baseline hematuria levels. Despite the cardinal diagnostic importance of hematuria at baseline in IgAN, only a few studies have analyzed its influence on the outcome of the disease, and the results have been discordant^38–41^. Nagai et al. reported higher 2-year eGFR slope and improved eGFR trajectory after methylprednisolone pulse therapy in patients with greater baseline hematuria whereas eGFR was stable in those with relatively mild hematuria^42^. A recent meta-analysis reported that baseline macroscopic hematuria was associated with a decreased risk of ESKD whereas baseline microscopic hematuria was associated with an increased risk for ESKD^43^. Overall, isolated hematuria at the time of biopsy might have increased the sensitivity for early detection of IgAN. The non-significant association between corticosteroid use and kidney outcomes in patients with lesser hematuria noted in this study may provide a clue to avoiding overtreatment with CS in the clinical management of IgAN^3^. However, this issue should be examined in future studies.

Two glomerular acute lesions including endocapillary hypercellularity and crescent formation statistically influenced the response to CS and CS + Tx. Similar results were observed in a VALIGA sub-study and a Tokyo cohort sub-study^6,7^. Implications of those two acute glomerular lesions may be limited by that of microscopic hematuria as some previous studies have reported an association between baseline microscopic hematuria and those pathological glomerular acute lesions^42,44^. Future studies with pathological data from repeated kidney biopsy after corticosteroid therapy may help identify the effects of corticosteroid therapy on pathological changes in patients with IgAN.

This study has several limitations. Despite robust statistical techniques due to the nature of the observational study, possibility of residual and unmeasured confounding remains. Regimens for corticosteroid therapy and tonsillectomy, adverse events after initial treatment and relapse/recurrence during follow-up might affect kidney prognosis. Subsequent studies to investigate those issues are planned. Longer follow-up data from observational studies are required to assess whether the effects of initial corticosteroid therapy are sustained over time. The present cohort included only Japanese patients and data from Japanese clinical practices. Racial differences in the susceptibility genes for IgAN have been suggested in genome-wide association studies^31,45^. East Asia is known as a region with patients with the highest susceptibility to IgAN. Therefore, the applicability of these findings to other populations is unknown.

In conclusion, a favorable kidney survival response to corticosteroid monotherapy and an even more favorable response with combined corticosteroid therapy and tonsillectomy was demonstrated in a nationwide prospective observational overlap-weighted cohort. More attention to baseline microscopic hematuria and glomerular acute lesions including endocapillary hypercellularity and crescent may improve the clinical practice of IgAN and may avoid overtreatment in corticosteroid-treated patients with IgAN.

## Methods

### Data sources and study population

J-IGACS is a nationwide, multicenter, electronic health record-based registry of patients with biopsy-proven primary IgAN described elsewhere^25^. Briefly, the registration period in J-IGACS was from April 1, 2005, to August 31, 2015. The database contains information on all inpatient and outpatient encounters, prescriptions, diagnostic codes, laboratory measurements, and pathologic data collected between April 1, 2005, and May 31, 2021. Clinical datasets were uploaded every 6 months to the J-IGACS website by each investigator. Patients were followed up from the date when kidney biopsy was performed until migration or departure from the practice or database, death, or the last date of data collection.

For the current analyses, we selected records of patients from J-IGACS database who had kidney pathological data and measurements of arterial BP, eGFR, proteinuria, and uRBC when kidney biopsy was performed and at least one follow-up measure of kidney outcomes.

The study was approved by the Ethical Committee of the Jikei University School of Medicine (16–174 [4402]) and each local ethics committee where participants were enrolled. The current study was performed according to the Declaration of Helsinki and adhered to STROBE reporting guidelines. All patients and/or parents/guardians provided written informed consent before enrollment in J-IGACS.

### Exposures

The exposure of interest was corticosteroid treatments comprising two types: CS and CS + Tx. Both were defined by initiation within 1 year after kidney biopsy. Each of the two exposure groups was compared to a group of patients who did not receive corticosteroid treatment within 1 year after kidney biopsy (non-CS). In further analyses, the CS + Tx group was compared with the CS group. Thus, the initial treatment categories included CS, CS + Tx, and non-CS, following intention-to-treat approach. Although the regimen of corticosteroid administration depended on each institute, principle one was one of two regimens: the Italian method and the Sendai method^8,46^. Both regimens involved corticosteroid pulse therapy, although the treatment duration varied between them (six versus twelve months).

### Kidney outcome

For patients aged ≥ 20 years when kidney biopsy was performed, the primary outcome was a composite endpoint of dialysis induction or a ≥ 50% increase in serum creatinine from baseline (confirmed by subsequent measurements)^8,17,47^. For patients < 20 years of age when kidney biopsy was performed, the primary outcome was a composite endpoint of dialysis induction or a reduction in eGFR of ≥ 25% (confirmed by subsequent measurement). Patients were followed up from the date when kidney biopsy was performed until the occurrence of the outcome of interest or the last examination date.

### Kidney function and other measurements

Serum creatinine and spot urine specimens were collected for each participant. Serum creatinine was assayed using an enzymatic method. In patients aged ≥ 20 years, eGFR was derived using the Chronic Kidney Disease Epidemiology Collaboration (CKD-EPI) equation modified by a Japanese coefficient^48^. In patients aged < 20 years at the time of kidney biopsy, eGFR was calculated using Uemura’s equation, and their follow-up eGFR measurements, collected at age > 20 years, were calculated using the revised CKD-EPI equation for Japanese adults^49^. The following equation was used to estimate mean arterial BP (MAP): MAP (mmHg) = 1/3 × pulse pressure + diastolic BP. The magnitude of microscopic hematuria was classified into two groups by its median value in the overall population. Regarding histological variables, we used the Oxford classification method (MEST-C score)^50,51^. The MEST-C score includes mesangial hypercellularity (M, M0/M1), endocapillary hypercellularity (E, E0/E1), segmental sclerosis (S, S0/S1), tubular atrophy/interstitial fibrosis (T, T0/T1 + 2), and crescents (C, C0/C1 + 2). The collected virtual slides were analyzed by five pathologists who were blinded to the clinical data. Final determinations of MEST-C scores were based on the agreement of three or more pathologists. Regarding renin–angiotensin–aldosterone system inhibitors (RAASi) use, if patients had already received RAASi at the time of kidney biopsy, such therapies with RAASi were also recorded as an initial prescription.

### Statistical analysis

Descriptive statistics are presented as means and standard deviations (SDs), median and interquartile range (IQR), and numbers and proportions as appropriate. Incidence rates for the primary outcome were calculated as the frequency of the outcome per 1,000 person-years of follow-up.

A multinomial logistic regression model was used to estimate the generalized propensity score of treatment with CS or CS + Tx compared with non-CS, conditional on patient covariates measured at baseline^52^. In total, 25 baseline covariates, which were grouped into covariate set 1 and covariate set 2, were chosen a priori as potential confounders and included in the propensity score models. There were 13 variables in covariate set 1: age, gender, diabetes mellitus, MAP, eGFR, proteinuria, uRBC, five types of MEST-C scores, and RAASi use. Covariate set 2 included 12 variables: hypertension, past history of macrohematuria, body mass index, serum uric acid, serum IgA, serum complement 3, Japanese clinical grading score^53^, Japanese histological grading score^54^, antiplatelet use, registered year, registered hospital area, and category of registered hospital (university or local leading one). Covariate set 1 had no missing data. A multivariate singular value decomposition imputation method was used for selected variables in covariate set 2 if data were missing.

An overlap-weighting approach was then used to construct a weighted cohort of patients receiving CS, CS + Tx, and non-CS^52,55^. Balance in baseline covariates among CS, CS + Tx, and non-CS groups before and after overlap weighting was assessed using a measure of absolute standardized difference (ASD), where a standardized difference of ≤ 0.1 was considered to ensure balance between groups^52,56^. Two types of ASD were measured: max of the pairwise ASDs (MASD) and all of the pairwise ASDs (PASD).

The weighted cumulative incidence rate of the primary kidney outcome according to the initial treatment categories was evaluated using Kaplan–Meier analysis. The weighted incidence rate in each group was calculated as the weighted number of incident events divided by the weighted overall number of 1000 person-years at risk. For the main analysis, weighted Cox proportional hazards models were used to estimate the weighted hazard ratios (HRs) for the study outcome in comparisons of CS with non-CS, CS + Tx with non-CS, and CS + Tx with CS. The consistency of findings observed in the main analysis was assessed by the test to determine the interaction *p* values within each stratum of baseline characteristics^57^. Multivariate Cox proportional hazard modeling adjusted with all variables in covariate set 1, except for stratifying factor, was performed in this subgroup analysis; however, overlap weighting was used to estimate survival curves according to initial treatment and incidence rate of kidney outcomes in each stratum per 1000 patient-years. A statistically significant interaction was defined as *p* value for interaction < 0.1. All statistical analyses were performed using R 4.2.1 with package PSweight, JMP13.2.0 (SAS Institute, Inc, Cary, NC), and STATA MP16.1 (STATA Corp.). Statistical significance was defined as a two-sided *p* < 0.05.

### Sensitivity analysis

Different inverse probability treatment weighting (IPW) analyses were employed in sensitivity analysis. Patients were weighted to derive the average treatment effect in the population that received CS + Tx (IPW-ATT) and the average treatment effect in the non-CS group (IPW-ATU)^52^. To assess the potential effects of unmeasured confounders on the identified associations between the exposures and the primary outcome, *E*-values were calculated^58^.

### Supplementary Information

Supplementary Information.

## Data Availability

The data that support the findings of this study are available on request from the corresponding author. The data are not publicly available due to ethical restrictions.
