# Supplementary material for: Associations of corticosteroid therapy and tonsillectomy with kidney survival in a multicenter prospective study for IgA nephropathy
Source: Sci Rep. 2023 Oct 27;13:18455. doi: 10.1038/s41598-023-45514-4 (PMC10611761; doi:10.1038/s41598-023-45514-4)
Supplement: Supplementary file 1 — Supplementary Information. [file 41598_2023_45514_MOESM1_ESM.docx]

**Supplementary Table of contents**

This article contains the following supplemental material online:

**Supplementary Table S1.** J-IGACS institutions.

**Supplementary Table S2a.** Variables having missing data in covariate set 2 by initial treatment before imputation, and their imputed data.

**Supplementary Table S2b.** Baseline characteristics in covariate set 2 by initial treatment category in unweighted and weighted cohorts.

**Supplementary Table S3.** Baseline characteristics in covariate set 1 by overlap weighting, IPW-ATT, and IPW-ATU.

**Supplementary Table S4.** Distribution of MEST-C score in various studies regarding corticosteroid treatment.

| **Supplementary Table S1. J-IGACS institutions** | | |
| --- | --- | --- |
| Region in Japan | Institutions, administrative divisions | University or local leading hospital |
| Eastern | Asahikawa Medical University Hospital, Hokkaido | University hospital |
|  | Japan Community Health Care Organization Sendai Hospital, Miyagi | Local leading hospital |
|  | Yamagata University Hospital, Yamagata | University hospital |
|  | Gunma University Hospital, Gunma | University hospital |
|  | Jichi Medical University Hospital, Tochigi | University hospital |
|  | Japanese Red Cross Ashikaga Hospital, Tochigi | Local leading hospital |
|  | University of Tsukuba Hospital, Ibaraki | University hospital |
|  | Tokyo Medical University Ibaraki Medical Center, Ibaraki | University hospital |
|  | The Jikei University School of Medicine Kashiwa Hospital, Chiba | University hospital |
|  | Dokkyo Medical University Saitama Medical Center, Saitama | University hospital |
|  | Kawaguchi Municipal Medical Center, Saitama | Local leading hospital |
|  | Tokyo Women's Medical University Hospital, Tokyo | University hospital |
|  | Tokyo Women's Medical University Hospital, Pediatrics, Tokyo | University hospital |
|  | Juntendo University Hospital, Tokyo | University hospital |
|  | The Jikei University School of Medicine Hospital, Tokyo | University hospital |
|  | The Jikei University School of Medicine Katsushika Medical Center, Tokyo | University hospital |
|  | The Jikei University School of Medicine Daisan Hospital, Tokyo | University hospital |
|  | Nippon Medical School Hospital, Tokyo | University hospital |
|  | Showa University Hospital, Tokyo | University hospital |
|  | Teikyo University Hospital, Tokyo | University hospital |
|  | Tokyo Metropolitan Children's Medical Center, Tokyo | Local leading hospital |
|  | St. Marianna University School of Medicine Hospital, Kanagawa | University hospital |
|  | Tokai University Hospital, Kanagawa | University hospital |
|  | Niigata University Medical & Dental Hospital, Niigata | University hospital |
| Western | Nagoya University Hospital, Aichi | University hospital |
|  | Fujita Health University Hospital, Aichi | University hospital |
|  | Kanazawa University Hospital, Ishikawa | University hospital |
|  | National Hospital Organization Kanazawa Medical Center, Ishikawa | Local leading hospital |
|  | Kyoto University Hospital, Kyoto | University hospital |
|  | Osaka City University Hospital, Osaka | University hospital |
|  | Kitano Hospital, Osaka | Local leading hospital |
|  | National Hospital Organization Osaka Medical Center, Osaka | Local leading hospital |
|  | Toyonaka Municipal Hospital, Osaka | Local leading hospital |
|  | Kobe University Hospital, Hyougo | University hospital |
|  | Shimane University Hospital, Shimane | University hospital |
|  | Tottori University Hospital, Tottori | University hospital |
|  | National Hospital Organization Kyushu Medical Center, Fukuoka | Local leading hospital |
|  | National Hospital Organization Fukuokahigashi Medical Center, Fukuoka | Local leading hospital |
|  | Japanese Red Cross Fukuoka Hospital, Fukuoka | Local leading hospital |
|  | Fukuoka University Hospital, Fukuoka | University hospital |
|  | Kyushu University Hospital, Fukuoka | University hospital |
|  | Miyazaki University Hospital, Miyazaki | University hospital |
|  | Nagasaki University Hospital, Nagasaki | University hospital |
|  | Tokushima University Hospital, Tokushima | University hospital |
| J-IGACS, Japan IgA Nephropathy Prospective Cohort Study. | | |

| **Supplementary Table S2a. Variables with missing data in covariate set 2 by initial treatment before imputation, and their imputed data.** | | | | | | | |
| --- | --- | --- | --- | --- | --- | --- | --- |
| Characteristics | | Before imputation | | | After imputation | | |
|  |  | non-CS | CS | CS+T | non-CS | CS | CS+Tx |
| No. of patients | | 338 | 239 | 364 | 338 | 239 | 364 |
| Body mass index | Value | 22.3±3.6 | 22.2±3.9 | 22.1±3.9 | 22.3±3.6 | 22.3±3.9 | 22.1±3.9 |
|  | No. missing | 8 (2.4) | 3 (1.3) | 3 (0.8) | 0 (0.0) | 0 (0.0) | 0 (0.0) |
| Serum uric acid | Value, mg/dl | 5.9±1.7 | 6.0±1.8 | 5.7±1.5 | 5.9±1.7 | 6.0±1.8 | 5.7±1.5 |
|  | No. missing | 11 (3.3) | 5 (2.1) | 7 (1.9) | 0 (0.0) | 0 (0.0) | 0 (0.0) |
| Serum IgA | Value, mg/dl | 324±120 | 350±137 | 325±112 | 325±116 | 348±134 | 326±110 |
|  | No. missing | 31 (9.2) | 13 (5.4) | 23 (6.3) | 0 (0.0) | 0 (0.0) | 0 (0.0) |
| Serum C3 | Value, mg/dl | 100±17 | 105±22 | 102±19 | 101±17 | 105±21 | 103±18 |
|  | No. missing | 51 (15.1) | 26 (10.9) | 59 (16.2) | 0 (0.0) | 0 (0.0) | 0 (0.0) |
| Values are shown as mean ± SD and number (%).  non-CS, non-corticosteroid use; CS, corticosteroid monotherapy; CS+Tx, corticosteroid therapy combined with tonsillectomy. | | | | | | | |

| **Supplementary Table S2b. Baseline characteristics in covariate set 2 by initial treatment category in unweighted and weighted cohorts.** | | | | | | | | |
| --- | --- | --- | --- | --- | --- | --- | --- | --- |
| Characteristics | Unweighted | | | | Weighted | | | |
|  | non-CS | CS | CS+Tx | MASD | non-CS | CS | CS+Tx | MASD |
| No. of patients | 338 | 239 | 364 |  | 314.5 | 314.5 | 314.5 |  |
| Hypertension | 138 (40.8) | 100 (41.8) | 87 (23.9) | 0.38 | 127.6(40.6) | 118.9 (37.8) | 113.5 (36.1) | 0.09 |
| Macrohematuria | 87 (25.7) | 63 (26.4) | 103 (28.3) | 0.06 | 73.0 (23.2) | 87.6 (27.9) | 82.7 (26.3) | 0.11 |
| Body mass index | 22.3±3.6 | 22.3±3.9 | 22.1±3.9 | 0.07 | 22.3±3.9 | 22.4±3.9 | 22.2±3.9 | 0.05 |
| serum uric acid, mg//dl | 5.9±1.7 | 6.0±1.8 | 5.7±1.5 | 0.17 | 5.9±1.6 | 6.0±1.8 | 5.8±1.5 | 0.11 |
| serum IgA, mg/dl | 325±116 | 348±134 | 326±110 | 0.19 | 337±128 | 338±121 | 325±107 | 0.11 |
| serum C3, mg/dl | 101±17 | 105±21 | 103±18 | 0.08 | 101±18 | 103±20 | 102±19 | 0.08 |
| CG score I | 201 (59.5) | 71 (29.7) | 146 (40.1) | 0.63 | 136.5 (43.4) | 123.2 (39.2) | 142.7 (45.4) | 0.13 |
| CG score II | 65 (19.2) | 99 (41.4) | 149 (40.9) | 0.48 | 101.7 (32.3) | 109.1 (34.7) | 104.9 (33.4) | 0.05 |
| CG score III | 72 (21.3) | 69 (28.8) | 69 (19.0) | 0.11 | 76.2 (24.2) | 82.1 (26.1) | 66.8 (21.2) | 0.11 |
| HG score I | 227 (67.2) | 135 (56.5) | 223 (61.3) | 0.22 | 185.5 (59.0) | 190.8 (60.7) | 198.8 (63.2) | 0.09 |
| HG score II | 68 (20.1) | 67 (28.0) | 99 (27.2) | 0.18 | 83.1 (26.4) | 80.2 (25.5) | 75.9 (24.1) | 0.05 |
| HG score III+IV | 43 (12.7) | 37 (15.5) | 42 (11.5) | 0.12 | 45.8 (14.6) | 43.5 (13.8) | 39.8 (12.6) | 0.06 |
| Anti-platelet use | 145 (42.9) | 99 (41.4) | 128 (35.2) | 0.16 | 129.7 (41.3) | 135.7 (43.2) | 128.1 (40.7) | 0.05 |
| Registration |  |  |  |  |  |  |  |  |
| Period 1 | 92 (27.2) | 54 (22.6) | 43 (11.8) | 0.11 | 65.3 (20.8) | 72.1 (22.9) | 71.9 (22.9) | 0.05 |
| Period 2 | 117 (34.6) | 71 (29.7) | 147 (40.4) | 0.23 | 122.3 (38.9) | 110.5 (35.1) | 109.4 (34.8) | 0.09 |
| Period 3 | 50 (14.8) | 39 (16.3) | 86 (23.6) | 0.23 | 54.7 (17.4) | 57.7 (18.4) | 58.3 (18.5) | 0.03 |
| Period 4 | 79 (23.4) | 75 (31.4) | 88 (24.2) | 0.18 | 72.1 (22.9) | 74.1 (23.6) | 74.9 (23.8) | 0.02 |
| Eastern-Hp. | 234 (69.2) | 137 (57.3) | 252 (69.2) | 0.25 | 205.4 (65.3) | 199.5 (63.5) | 198.7 (63.2) | 0.05 |
| University Hp. | 269 (79.6) | 59 (24.7) | 316 (86.8) | 0.29 | 243.4 (77.4) | 254.7 (81.0) | 254.6 (81.0) | 0.09 |
| Unweighted or weighted values are shown as number (%), mean ± SD.  No., number; CG, Japanese clinical grading score; HG, Japanese histological grading score; Period 2, 2010–2011-year; Period 3, 2012-year; Period 4, 2013-2015-year; Eastern-Hp., hospital located in eastern Japan (**Supplemental table 1**); Hp., hospital; non-CS, non-corticosteroid use; CS, corticosteroid monotherapy; CS+Tx, corticosteroid therapy combined with tonsillectomy; MASD, max of the pairwise absolute standardized difference. | | | | | | | | |

| **Supplementary Table S3. Baseline characteristics in covariate set 1 by overlap weighting, IPW-ATT, and IPW-ATU** | | | |
| --- | --- | --- | --- |
|  | Main analysis | Sensitivity analyses | |
|  | Overlap | IPW-ATT | IPW-ATU |
| No. of patients | 943.4 | 1092.0 | 1014.0 |
| Age, years | 39.5 ± 16.5 | 34.9 ± 15.3 | 43.0 ± 17.1 |
| Age < 20 years ^a^ | 94.3 (10.0) | 155.5 (14.2) | 70.1 (6.9) |
| Woman | 478.7 (50.7) | 599.5 (54.9) | 496.1 (48.9) |
| DM | 24.2 (2.6) | 18.5 (1.7) | 17.8 (1.8) |
| MAP, mmHg | 90.6 ± 13.9 | 88.8 ± 13.6 | 91.2 ± 13.6 |
| MAP ≥ 90 mmHg ^a^ | 472.7 (50.1) | 491.7 (45.0) | 522.7 (51.5) |
| eGFR, ml/min/1.73 m^2^ | 74.2 ± 28.1 | 79.8 ± 28.7 | 72.9 ± 27.8 |
| eGFR < 60 ml/min/1.73 m^2 a^ | 291.7 (30.9) | 266.2 (24.4) | 352.6 (34.8) |
| Proteinuria, g/day | 0.60 (0.30–1.21) | 0.64 (0.32–1.25) | 0.43 (0.19–0.86) |
| Proteinuria > 1.0 g/day ^a^ | 308.5 (32.7) | 378.7 (34.7) | 219.0 (21.6) |
| Hematuria ≥ 20/HPF | 488.4 (51.8) | 603.4 (55.3) | 444.3 (43.8) |
| RAASi use | 551.9 (58.5) | 592.4 (54.3) | 653.5 (64.5) |
| MEST-C score |  |  |  |
| M1 | 295.2 (31.3) | 317.4 (29.1) | 256.3 (25.3) |
| E1 | 362.0 (38.4) | 443.2 (40.6) | 240.2 (23.7) |
| S1 | 714.9 (75.8) | 867.7 (79.5) | 660.9 (65.2) |
| T1+2 | 209.8 (22.2) | 204.8 (18.8) | 220.1 (21.7) |
| C1+2 | 368.2 (39.0) | 510.8 (46.8) | 230.4 (22.7) |
| Initial treatment |  |  |  |
| non-CS | 314.5 (33.3) | 364.0 (33.3) | 338.0 (33.3) |
| CS | 314.5 (33.3) | 364.0 (33.3) | 338.0 (33.3) |
| CS+Tx | 314.5 (33.3) | 364.0 (33.3) | 338.0 (33.3) |
| Weighted values are shown as number (%), mean ± SD, or median (IQR).  IPW, inverse probability treatment weighting analysis; ATT, average treatment effect in the treated as corticosteroid therapy combined with tonsillectomy group; ATU, average treatment effect in the untreated as non-corticosteroid use; DM, diabetes mellitus; MAP, mean arterial blood pressure; eGFR, estimated glomerular filtration rate; HPF, high-power field; RAASi, renin-angiotensin-aldosterone system inhibitor; M, mesangial hypercellularity score; E, endocapillary hypercellularity score; S, segmental sclerosis score; T, tubulointerstitial fibrosis/atrophy score; C, crescent score.  ^a^ neither included in covariate set 1 nor employed for propensity score but shown here to promote understanding of the cohort. | | | |

| **Supplementary Table S4. Distribution of MEST-C score in various studies regarding corticosteroid treatment.** | | | | | | | | | |
| --- | --- | --- | --- | --- | --- | --- | --- | --- | --- |
| Study category | Study name | Baseline characteristics | | | | | | Favorable corticosteroid therapy in kidney survival | References |
|  |  | Majority in race/ethnicity | M1, % | E1, % | S1, % | T1+2, % | C1+2, % |  |  |
| RCT | TESTING | Chinese,  South Asian | 60 | 25 | 68 | 51 | 56 | yes | 4, 5 |
|  | STOP-IgAN | European | 26 | 17 | 91 | 41 | 31 | no | 3, 26 |
| Matched or weighted study | VALIGA | European | 17 | 6 | 41 | 15 | 50 | yes | 32, 43 |
|  | Tokyo | Japanese | 46 | 56 | 77 | 29 | 60 | yes | 7 |
|  | J-IGACS | Japanese | 31 | 38 | 76 | 22 | 39 | yes | Present study |
| M, mesangial hypercellularity; E, endocapillary hypercellularity; S, segmental sclerosis; T, tubular atrophy/interstitial fibrosis; C, crescent; RCT randomized controlled study. | | | | | | | | | |
